# Preoperative anti-VEGF and the cumulative risk of post-operative vitreous hemorrhage in PDR: a 2-year survival analysis and evaluation of surgical burden

**DOI:** 10.1186/s40942-026-00871-w

**Published:** 2026-05-29

**Authors:** Yuxian Lin, Ruibin Wu, Gengjia Li

**Affiliations:** Department of Ophthalmology, The First Affiliated Hospital of Shantou University Medical College, No. 57 Changping Road, Shantou, 515041 China

**Keywords:** Proliferative diabetic retinopathy, Vitreous hemorrhage, Anti-VEGF, Reoperation, Survival analysis, Surgical burden

## Abstract

**Purpose:**

To investigate the long-term risk factors for post-operative vitreous hemorrhage (VH) within 24 months following vitrectomy for proliferative diabetic retinopathy (PDR), evaluate the protective efficacy of preoperative anti-VEGF therapy, and quantify the associated secondary surgical burden.

**Methods:**

This retrospective cohort study included 735 eyes from 735 PDR patients. A 24-month survival analysis was employed to categorize patients into VH (*n* = 179) and non-VH (*n* = 556) groups. Independent predictors were identified using univariate and multivariate Cox proportional hazards models. A sensitivity analysis was performed on a “pure hemorrhage model” (*n* = 676) by excluding eyes with post-operative tractional retinal detachment (TRD) to isolate the direct vascular-stabilizing effect of anti-VEGF.

**Results:**

The overall cumulative incidence of post-operative VH was 18.4% at 12 months and 25.7% at 24 months. Multivariate Cox analysis revealed that preoperative anti-VEGF injection emerged as the strongest protective factor, associated with a 63% reduction in VH hazard (HR 0.37; 95% CI, 0.27–0.51; *P* < 0.001). This protective effect remained robust in the sensitivity analysis (HR 0.32; *P* < 0.001), which is consistent with a potential vascular-stabilizing effect independent of anatomical success. Independent risk factors included severe fibrovascular traction (HR 1.93; *P* = 0.003), younger age, higher HbA1c, and elevated serum creatinine (all *P* < 0.05). Patients in the VH group had significantly poorer final visual outcomes (1.33 ± 0.75 vs. 0.92 ± 0.60 LogMAR; *P* < 0.001) and a markedly higher reoperation rate (31.3% vs. 7.2%). Notably, hemorrhage-related indications accounted for 58.4% of the total secondary surgical workload.

**Conclusion:**

Post-operative VH is a dominant driver of long-term visual impairment and secondary surgical burden in PDR. Preoperative anti-VEGF supports the hypothesis that preoperative anti-VEGF may contribute to sustained microvascular stabilization. Vitreous hemorrhage-related indications accounted for 58.4% of secondary surgical interventions, suggesting that clinical strategies aimed at reducing recurrent VH could meaningfully decrease the overall reoperation burden in this population.

**Supplementary Information:**

The online version contains supplementary material available at 10.1186/s40942-026-00871-w.

## Introduction

Proliferative diabetic retinopathy (PDR) remains a leading cause of irreversible vision loss worldwide [1, 2]. While pars plana vitrectomy (PPV) has revolutionized the management of PDR-related complications, post-operative vitreous hemorrhage (VH) remains a significant challenge, complicating post-surgical recovery for both clinicians and patients [3, 4]. Despite advancements in surgical instrumentation and techniques, the reported incidence of recurrent VH varies widely, ranging from 10% to over 70% [3, 5–7], often leading to delayed visual rehabilitation and an increased requirement for secondary surgical interventions.

Historically, most clinical investigations have focused on early post-operative VH (occurring within 4 weeks), attributing it to residual blood or intraoperative maneuvers [8, 9]. However, the long-term clinical course and cumulative risk of VH over a multi-year horizon remain poorly characterized [10, 11]. Furthermore, while preoperative anti-vascular endothelial growth factor (VEGF) therapy is widely employed to facilitate surgical dissection and reduce intraoperative bleeding, its long-term protective efficacy and underlying mechanisms remain subjects of active debate [12, 13]. Specifically, it remains unclear whether the reduced risk of VH associated with anti-VEGF is merely a byproduct of improved anatomical success (i.e., prevention of tractional retinal detachment, TRD) or a direct, sustained stabilization of the retinal microvasculature.

A critical gap in the current literature is the lack of large-scale studies using a standardized long-term survival framework that accounts for systemic metabolic factors, surgical complexity, and the dynamic nature of neovascularization [11, 14]. Moreover, the quantifiable impact of recurrent VH on the overall secondary surgical burden—a key metric for healthcare resource allocation—has not been sufficiently established in large-scale longitudinal cohorts [15].

In this study, we conducted a 24-month survival analysis on a cohort of 735 PDR patients to identify independent predictors of long-term post-operative VH. By employing a multivariate Cox proportional hazards model and a unique sensitivity analysis that decoupled VH from anatomical failure (TRD), we aimed to: (1) delineate the cumulative incidence and temporal trends of VH over a 2-year period; (2) evaluate the potential vascular-stabilizing association of preoperative anti-VEGF; and (3) quantify the clinical and surgical burden attributable to recurrent hemorrhage. Our findings provide a robust evidence-based framework for optimizing perioperative management and improving long-term prognosis in PDR surgery.

## Methods

### Study design and ethical approval

This single-center, retrospective cohort study was conducted at the Department of Ophthalmology, The First Affiliated Hospital of Shantou University Medical College, in adherence to the tenets of the Declaration of Helsinki. The study protocol was approved by the Institutional Review Board of the First Affiliated Hospital of Shantou University Medical College (Approval ID: B-2025-212).

### Patient selection and grouping

A total of 1,325 patients with PDR who underwent pars plana vitrectomy (PPV) or combined phaco-vitrectomy (PPV&P) between January 2019 and January 2025 were screened. Exclusion criteria included: (1) prior vitrectomy or cataract surgery in the study eye; (2) baseline rubeosis iridis or neovascular glaucoma (NVG); (3) confounding ocular pathologies (e.g., age-related macular degeneration, high myopia with axial length > 26 mm, or retinal vascular occlusions); (4) intraoperative complications such as posterior capsule tear; and (5) incomplete follow-up or missing systemic markers.

**Fig. 1:**
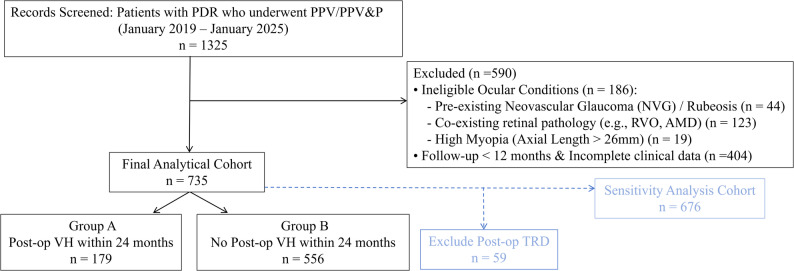
Flowchart of patient enrollment and study design. A total of 1325 patients with proliferative diabetic retinopathy (PDR) who underwent pars plana vitrectomy (PPV) or combined phaco-vitrectomy (PPV&P) were initially screened. After excluding 186 cases with ineligible ocular conditions and 404 cases with insufficient follow-up or incomplete data, 735 patients were included in the final analytical cohort. These patients were categorized into Group A (Post-operative VH within 24 months, *n* = 179) and Group B (No post-operative VH within 24 months, *n* = 556). To evaluate the independent effect of risk factors, a sensitivity analysis was performed on a subset of 676 patients by excluding those who developed post-operative tractional retinal detachment (TRD). Abbreviations: VH: vitreous hemorrhage; PDR: proliferative diabetic retinopathy; NVG: neovascular glaucoma; RVO: retinal vein occlusion; AMD: age-related macular degeneration; TRD: tractional retinal detachment

Ultimately, 735 eyes from 735 patients were included. Patients were stratified using a 24-month survival analysis: Group A: Developed post-operative VH within 24 months (*n* = 179). Group B: Remained VH-free for at least 24 months (*n* = 556) (Fig. 1). To ensure comparability, only VH events occurring within the first 24 months were used for primary grouping and analysis.

### Surgical procedures

All surgeries were performed by two experienced vitreoretinal surgeons following a standardized 23-gauge three-port PPV protocol. The procedure included core vitrectomy, meticulous membrane delamination and segmentation to relieve all fibrovascular traction, and comprehensive panretinal endophotocoagulation (PRP) extending to the ora serrata. Intravitreal tamponade (balanced salt solution, air, C3F8, or silicone oil) was selected at the surgeon’s discretion. Preoperative anti-VEGF injections (Conbercept or Ranibizumab) were administered 2–14 days before surgery in selected cases to facilitate membrane dissection and reduce intraoperative bleeding [12].

### Data collection and definitions

Preoperative systemic markers included HbA1c and serum creatinine. Surgical complexity was assessed using the Kroll et al. traction grading system, incorporating the extent of neovascularization and detachment range based on clinical examination and imaging modalities such as optical coherence tomography (OCT) and fundus photography [16].

The primary outcome was the cumulative incidence of post-operative VH, defined as any recurrent hemorrhage obscuring retinal details for over three weeks. Diagnosis was confirmed via slit-lamp biomicroscopy; for eyes with opaque media, B-scan ultrasonography was routinely employed to verify hemorrhage and exclude anatomical complications. VH events were further stratified into early (≤ 1 month) and late (> 1 month) recurrence.

Secondary outcomes included final best-corrected visual acuity (LogMAR), the incidence of post-operative tractional retinal detachment (TRD), and the rate of secondary surgical interventions (reoperation).

### Statistical analysis

Continuous variables were compared using independent-sample t-tests or Wilcoxon rank-sum tests; categorical variables were analyzed via Chi-squared or Fisher’s exact tests. Time-to-VH data were analyzed using the Kaplan-Meier method with Log-rank tests. Independent predictors were identified through univariate and multivariate Cox proportional hazards models using a backward stepwise approach.

To rigorously address potential selection bias and confounding factors inherent in the retrospective design, a three-tier validation analysis was implemented:

Baseline Comparability: Pearson’s chi-squared tests were used to assess whether preoperative traction severity influenced the decision to administer anti-VEGF.

Interaction & Confounding Analysis: Interaction terms between anti-VEGF treatment and traction grade were incorporated into the Cox models to assess efficacy consistency. Additionally, post-operative TRD was evaluated as an exploratory, macroscopic surrogate for ‘incomplete traction release’ to partially adjust for the impact of anatomical success on long-term vascular outcomes, although we recognize that TRD cannot fully represent overall intraoperative thoroughness.

Sensitivity & Subgroup Analysis: A sensitivity analysis was performed by excluding all patients with post-operative TRD (*n* = 676) to isolate the vascular-stabilizing effect. Subgroup analyses were also conducted across traction levels to verify the robustness of the protection.

Model discrimination was assessed using Harrell’s C-index. Analyses were performed using SPSS 25.0 and R (version 4.2.2). Two-sided *P* < 0.05 was considered statistically significant.

## Results

### Baseline characteristics and systemic profiles

A total of 735 eyes from 735 patients with PDR were analyzed. Based on the 24-month survival analysis, 179 eyes (24.4%) experienced post-operative vitreous hemorrhage (VH) (Group A), while 556 eyes (75.6%) remained VH-free (Group B). The mean follow-up was 31.4 months, with no significant difference between groups (*P* = 0.236), ensuring an unbiased observation window.

Patients in Group A were significantly younger (50.6 ± 9.6 vs. 53.4 ± 9.7 years; *P* = 0.001) and exhibited poorer metabolic control, including higher HbA1c (7.60 ± 1.41% vs. 7.31 ± 1.66%; *P* = 0.036) and elevated serum creatinine levels (114.96 ± 42.96 vs. 101.74 ± 69.38 µmol/L; *P* = 0.016).

Regarding preoperative ocular status, Group A had a significantly lower rate of preoperative anti-VEGF injection (64.2% vs. 85.4%; *P* < 0.001) and history of PRP (25.1% vs. 34.7%; *P* = 0.022). Furthermore, Group A presented with higher surgical complexity, characterized by more severe fibrovascular traction (Severe: 50.3% vs. 32.0%; *P* < 0.001) and a higher prevalence of combined TRD and VH as the primary surgical indication (42.5% vs. 30.8%; *P* = 0.012). Surgical procedures, including surgery type, use of endolaser, and intraoperative TA, were comparable between groups (all *P* > 0.05; Table 1).

**Table 1:** Baseline demographic and clinical characteristics of patients stratified by the occurrence of post-operative vitreous hemorrhage (VH) within 2 years

Parameter	No Post-op VH(*n* = 556)	Post-op VH(*n* = 179)	*P* Value
Age, mean (SD), y	53.40(9.67)	50.61(9.61)	**0.001**
Sex, No. (%)			
Male	365(65.6)	116(64.8)	0.908
Female	191(34.4)	142(35.2)	
History of diabetes, mean (SD), y	8.71(6.28)	8.27(5.77)	0.410
HbA1c, mean (SD),%	7.31(1.66)	7.60(1.41)	0.036
Creatinine, mean (SD), umol/L	101.74(69.38)	114.96(42.96)	0.016
Previous anti‑VEGF within 2 weeks, No. (%)	475(85.4)	115(64.2)	**<0.001**
Previous laser, No. (%)	193(34.7)	45(25.1)	**0.022**
Surgery indications, No. (%)			
VH	354(63.7)	97(54.2)	
TRD	31(5.6)	6(3.4)	**0.012**
TRD + VH	171(30.8)	76(42.5)	
Preoperative lens nuclear sclerosis, No. (%)			
Grade 0/1	344(61.9)	108(60.3)	
Grade 2	201(36.2)	64(35.8)	0.346
Grade 3/4	11(2.0)	7(3.9)	
Preoperative Grading of Traction, No. (%)			
Severe	178(32.0)	90(50.3)	
Mild or moderate	232(41.7)	59(33.0)	**<0.001**
No traction	146(26.3)	30(16.8)	
Surgery type, No. (%)			
PPV	459(82.6)	144(80.4)	0.598
PPV&P	97(17.4)	35(19.6)	
Surgery Procedures			
Endotamponade, No. (%)			
BSS	365(65.6)	104(58.1)	
Air	21(3.8)	6(3.4)	0.113
Silicone Oil	121(21.8)	55(30.7)	
C3F8 gas	49(8.8)	14(7.8)	
Endolaser, No. (%)	550(98.9)	177(98.9)	0.811
TA injection, No. (%)	272(48.9)	80(44.7)	0.369
Preoperative IOP, mean (SD), mmHg	13.24(1.65)	13.28(1.63)	0.799
Preoperative BCVA, Snellen(logMAR), mean (SD)	1.58(0.58)	1.59(0.53)	0.896
Follow-up time, mean (SD), y	31.67(10.52)	30.62(9.98)	0.236

Nuclear sclerosis was assessed at the slit lamp and classified as follows: Grade 0: Clear lens.Grade 1: Early nuclear sclerosis with mild yellow discoloration of the posterior lens in the slit beam.Grade 2: Yellow discoloration throughout the lens.Grade 3: Yellow-brown discoloration throughout the lens.Grade 4: Brown discoloration of the entire lens

Preoperative proliferative traction was graded as no traction(complete posterior vitreous detachment), mild (localized fibrosis without macular involvement), moderate (multifocal fibrosis with partial macular traction), or severe (extensive fibrosis with macular detachment or broad RD), based on clinical and imaging assessment, incorporating extent of neovascularization and detachment range

Continuous variables are presented as mean (standard deviation) and compared using the Student’s t-test or Wilcoxon rank-sum test. Categorical variables are presented as number (percentage) and compared using the Chi-square test or Fisher’s exact test

Abbreviations: HbA1c,Glycosylated hemoglobin;VH, vitreous hemorrhage; TRD, tractional retinal detachment; Anti-VEGF, anti-vascular endothelial growth factor; PPV, pars plana vitrectomy; PPV&P, combined phaco-vitrectomy and PPV; IOP, intraocular pressure; BCVA, best-corrected visual acuity; logMAR, logarithm of the minimum angle of resolution; TA, triamcinolone acetonide; BSS, balanced salt solution

**Fig. 2:**
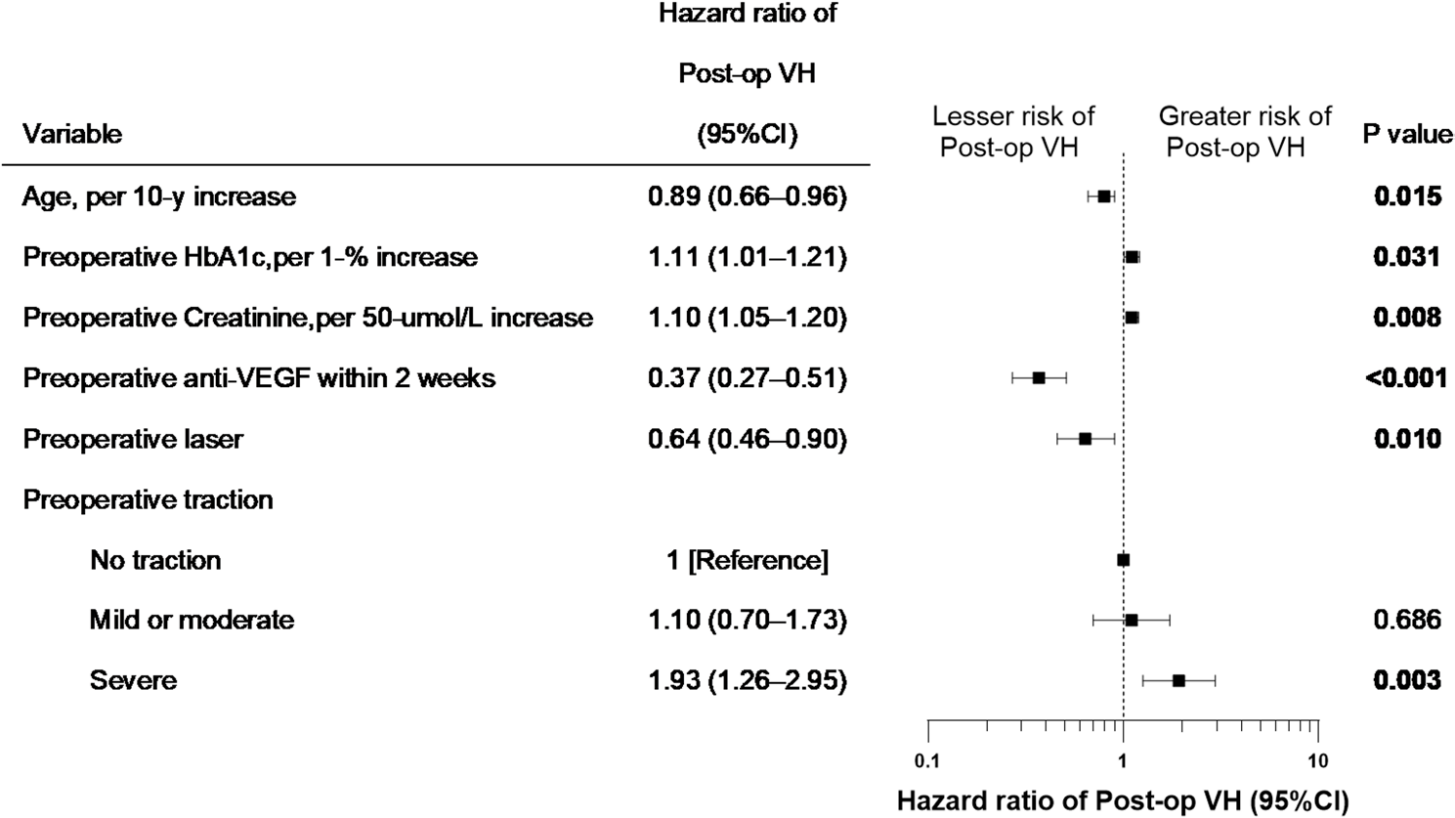
Forest plot of independent predictors for post-operative vitreous hemorrhage (VH) within 24 months. The forest plot displays the hazard ratios (HR) and 95% confidence intervals (CI) derived from the final multivariate Cox proportional hazards model. The vertical dashed line represents a hazard ratio of 1.0 (null effect). Abbreviations: HR, hazard ratio; CI, confidence interval; Anti-VEGF, anti-vascular endothelial growth factor; HbA1c, glycated hemoglobin

To ensure methodological rigor, we performed two validation analyses. First, stratified analysis within the anti-VEGF subgroup confirmed no disparities in baseline traits or prognosis between Conbercept and Ranibizumab (HR = 0.89,*P* = 0.553), justifying the pooling of agents (Supplementary Tables 1 & Fig. 1). Second, treatment allocation was balanced across traction grades (χ 2 = 1.09,*P* = 0.581), indicating no significant selection bias in anti-VEGF administration (Supplementary Tables 2 & Fig. 2).

### Independent predictors of post-operative VH

Univariate Cox regression identified younger age, higher HbA1c, elevated creatinine, severe traction, and silicone oil tamponade as risk factors, while preoperative anti-VEGF and laser were protective (Table 2).

**Table 2:** Univariate and multivariate cox proportional hazards analysis for post-operative VH within 24 months

Variable	Univariate HR (95% CI)	*P* Value	Multivariate HR (95% CI)	*P* Value
Demographics				
Age (per 10 year increase)	0.80 (0.60–0.90)	**0.001**	0.89 (0.66–0.96)	**0.015**
Sex (Female vs. Male)	1.06 (0.78–1.44)	0.704	-	-
Systemic Factors				
DM Duration (per year)	0.99 (0.97–1.01)	0.451	-	-
HbA1c (%)	1.10 (1.01–1.20)	**0.029**	1.11 (1.01–1.21)	**0.031**
Creatinine (per 50 µmol/L increase)	1.10 (1.05–1.20)	**0.033**	1.10 (1.05–1.20)	**0.008**
Preoperative Status				
Anti-VEGF within 2 weeks	0.37 (0.27–0.50)	**< 0.001**	0.37 (0.27–0.51)	**< 0.001**
Preoperative Laser	0.66 (0.47–0.92)	**0.015**	0.64 (0.46–0.90)	**0.010**
Surgery indications				
VH	Reference	-	Reference	-
TRD	0.71 (0.31–1.61)	0.411	-	-
TRD + VH	1.52 (1.13–2.05)	**0.006**	-	-
Traction Grade				
No traction	Reference	-	Reference	-
Mild or moderate	1.16 (0.75–1.81)	0.498	1.10 (0.70–1.73)	0.686
Severe	2.15 (1.42–3.25)	**< 0.001**	1.93 (1.26–2.95)	**0.003**
Surgical Factors				
Surgery Type (PPV vs. PPV&P)	0.79 (0.55–1.14)	0.212	-	-
Tamponade Type				
BSS	Reference	-	-	-
Air	0.89 (0.39–2.02)	0.774	-	-
C3F8 gas	0.97 (0.55–1.69)	0.904	-	-
Silicone Oil	1.55 (1.12–2.15)	**0.009**	-	-

Model Performance: The multivariate model demonstrated a good discriminative ability with a C-index of 0.68 (95% CI, 0.64–0.72)

Variable Selection: Candidate variables with *P* < 0.10 in the univariate analysis or those clinically relevant (e.g., Age) were entered into the final multivariate Cox proportional hazards model. A backward stepwise selection based on the Akaike Information Criterion (AIC) was employed to refine the model

Statistical Robustness: The proportional hazards assumption was checked using Schoenfeld residuals. Although the global test showed a minor deviation (*P* = 0.001), a sensitivity analysis using a stratified Cox model confirmed that the hazard ratios for the primary variable (Anti-VEGF) remained highly consistent (HR 0.37, *P* < 0.001). Multicollinearity was ruled out with all variance inflation factors (VIF) < 3.0

Abbreviations: HR, hazard ratio; CI, confidence interval; HbA1c, glycated hemoglobin; Anti-VEGF, anti-vascular endothelial growth factor; PPV, pars plana vitrectomy; PPV&P, phaco-vitrectomy

The multivariate Cox model exhibited moderate discrimination (C-index = 0.68), which supports robust independent statistical associations rather than establishing high individual predictive accuracy. The model indicated that preoperative anti-VEGF injection was the most potent protective factor, associated with a 63% reduction in VH hazard (HR 0.37; 95% CI, 0.27–0.51; *P* < 0.001). Preoperative laser also remained independently protective (HR 0.64; *P* = 0.010). Conversely, independent risk factors included severe traction (HR 1.93; *P* = 0.003), younger age (per 10-year increase: HR 0.89; *P* = 0.015), higher HbA1c (HR 1.11; *P* = 0.031), and elevated creatinine (per 50 µmol/L increase: HR 1.10; *P* = 0.008). A stratified Cox analysis confirmed the robustness of the anti-VEGF protective effect across different traction grades (HR 0.37; *P* < 0.001; Fig. 2).Subgroup analysis further confirmed that the protective effect of anti-VEGF remained consistent across all traction levels (Supplementary Table 2, Part C).

### Temporal trends and cumulative incidence

**Fig. 3:**
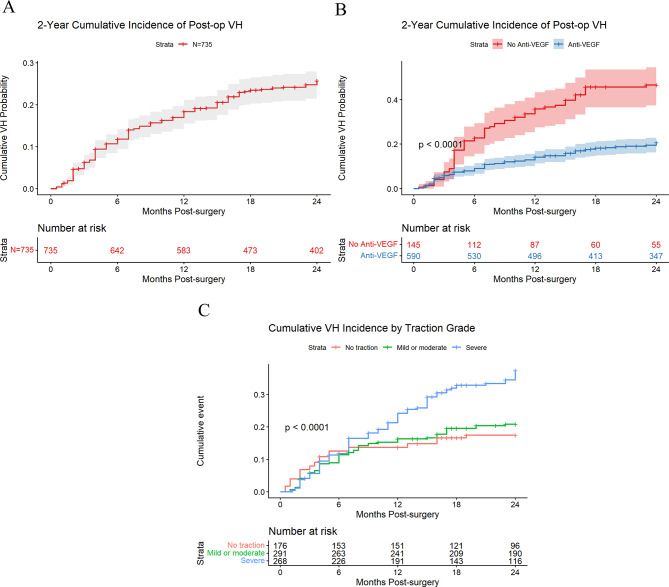
Cumulative incidence of post-operative vitreous hemorrhage (VH) during the 24-month follow-up period. (**A**) Overall Population (*N* = 735): The cumulative incidence of VH for the total cohort was 18.4% (95% CI: 15.5–21.2%) at 12 months, and 25.7% (95% CI: 22.3–28.9%) at 24 months. (**B**) Stratified by Preoperative Anti-VEGF: Patients who received preoperative anti-VEGF injection (blue line) showed a significantly lower cumulative incidence of VH compared to those without (red line; Log-rank *P* < 0.001). At 24 months, the risk in the non-anti-VEGF group (46.6%) was more than double that of the anti-VEGF group (20.7%). (**C**) Stratified by Tractional Severity: The cumulative hazard increased significantly with higher traction grades (Log-rank *P* < 0.001). Severe traction (blue line) exhibited the highest risk, reaching a 37.3% incidence at 24 months. Shaded areas represent 95% confidence intervals. The tables below the plots provide the “Number at risk” (eyes remaining VH-free) at each 6-month interval

Kaplan-Meier estimates revealed an overall cumulative VH incidence of 18.4% (95% CI: 15.5–21.2%) at 12 months and 25.7% (95% CI: 22.3–28.9%) at 24 months (Fig. 3A).

The protective impact of anti-VEGF was striking: the 24-month cumulative incidence in the anti-VEGF group was only 20.7%, compared to 46.6% in the non-injected group (Log-rank *P* < 0.001; Fig. 3B). Anatomical severity also dictated long-term risk; the severe traction group showed an accelerated cumulative incidence, rising from 24.2% at 12 months to 37.3% at 24 months, which was significantly higher than those with milder traction grades (Log-rank *P* < 0.001; Fig. 3C).

Distinguishing by timing, late VH (> 1 month) emerged as the primary driver of morbidity, exhibiting a significantly higher reoperation rate than early VH (32.4% vs. 11.1%, *P* < 0.001) and poorer visual outcomes (1.34 vs. 1.14 LogMAR; Supplementary Table 3). Notably, anti-VEGF therapy conferred potent protection specifically against these late-onset recurrences (OR = 0.29, *P* < 0.001).

### Sensitivity analysis: findings consistent with potential vascular stabilization

To isolate the direct pharmacotherapeutic effect of anti-VEGF from anatomical outcomes, we first confirmed that post-operative VH and TRD were statistically independent events (χ² = 0.0017, *P* = 0.967).In a “pure hemorrhage model” excluding all patients with post-operative TRD (*N* = 676), the protective effect of anti-VEGF remained highly robust (HR 0.32; 95% CI, 0.23–0.44; *P* < 0.001). Other factors, including age, laser history, and traction grade, maintained their predictive significance (Table 3). This consistency, alongside a moderate model discrimination (C-index = 0.696), supports the hypothesis that anti-VEGF potentially contributes to microvascular stability independent of anatomical success, further aligning with the concept of a sustained “vascular buffer” effect at the cohort level.

**Table 3:** Sensitivity analysis of risk factors for post-operative VH in patients without post-operative TRD (*N* = 676)

Variable	Hazard Ratio (95% CI)	z value	*P* Value
Demographics			
Age (per 10 year increase)	0.77 (0.62–0.93)	-2.77	**0.006**
Systemic Factors			
HbA1c (%)	1.10 (1.00–1.21)	1.94	0.052
Creatinine (per 50 µmol/L increase)	1.12 (1.02–1.22)	2.35	**0.019**
Ocular Factors			
Preoperative Anti-VEGF within 2 weeks	**0.32 (0.23–0.44)**	**-6.89**	**< 0.001**
Preoperative Laser	0.61 (0.43–0.87)	-2.73	**0.006**
Traction Grade			
No traction	Reference	-	-
Mild or moderate	1.15 (0.71–1.84)	0.57	0.572
Severe	2.11 (1.35–3.30)	3.27	**0.001**

Model Performance: The C-index for this sensitivity model was 0.696, indicating high discriminative power in the absence of post-operative TRD

Rationale: This sensitivity analysis was conducted to evaluate whether the protective effect of anti-VEGF was independent of its potential role in preventing anatomical failure (TRD)

Association Test: A Chi-square test confirmed that post-operative VH and post-operative TRD were independent events in this cohort (*X*² = 0.0017, *P* = 0.967)

Abbreviations: VH, vitreous hemorrhage; Anti-VEGF, anti-vascular endothelial growth factor; TRD, tractional retinal detachment; HR, hazard ratio; CI, confidence interval

To further explore the relationship between pharmacological efficacy and anatomical outcomes, an interaction model was constructed using post-operative TRD as a proximal indicator for traction management.Post-operative TRD did not emerge as an independent predictor of long-term VH (*P* = 0.913). The non-significant interaction terms (*P* = 0.224 and 0.866) indicated that the protective trends associated with anti-VEGF therapy remained stable across different anatomical outcomes, suggesting that the observed vascular benefit is not entirely confounded by macroscopic anatomical failure (Supplementary Table 4).

### Clinical consequences and surgical burden

**Fig. 4:**
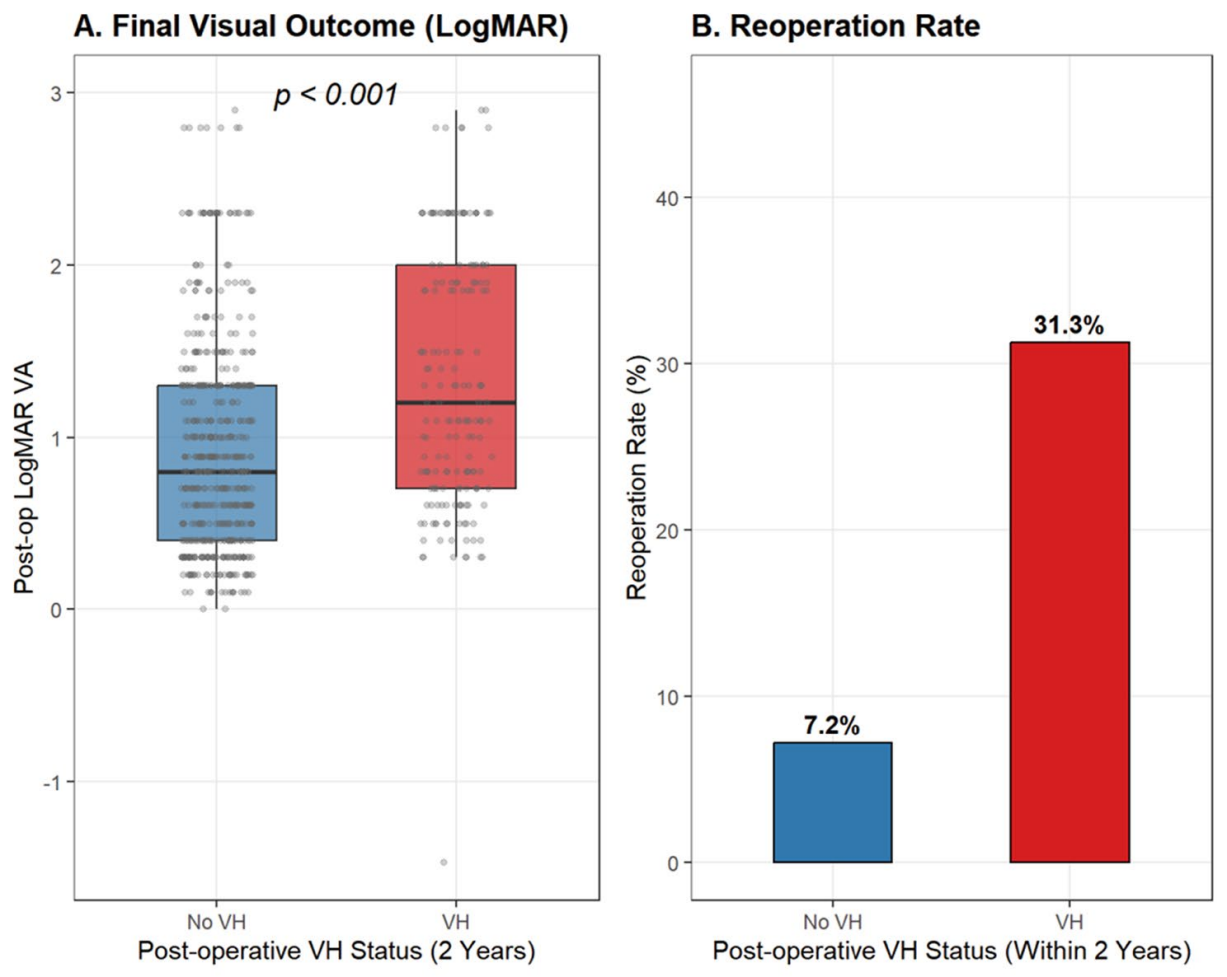
Impact of post-operative vitreous hemorrhage (VH) on visual recovery and surgical burden within 2 years. (**A**) Final Visual Outcome: Comparison of final LogMAR visual acuity (VA) between the VH and non-VH groups. Patients who experienced post-operative VH had significantly poorer visual outcomes (1.33 ± 0.75 vs. 0.92 ± 0.60; *p* < 0.001). Boxplots represent the median and interquartile range; whiskers represent the 1.5x IQR. (**B**) Reoperation Rate: Comparison of the incidence of secondary pars plana vitrectomy (PPV). The reoperation rate was more than four times higher in the VH group compared to the non-VH group (31.3% vs. 7.2%)

Post-operative VH was associated with significantly inferior functional recovery and increased surgical burden. Patients in Group A achieved a poorer final visual acuity (1.33 ± 0.75 vs. 0.92 ± 0.60 LogMAR; *P* < 0.001; Fig. 4A).

The reoperation rate was more than fourfold higher in the VH group compared to the non-VH group (31.3% vs. 7.2%; Fig. 4B). Among the 96 eyes (13.1% of total) requiring secondary PPV, recurrent VH alone was the leading indication (43.8%), followed by post-operative TRD (41.7%) and combined VH/TRD (14.6%; Table 4). Crucially, vitreous hemorrhage-related indications accounted for 58.4% of the total secondary surgical interventions, underscoring its predominant role in driving the post-operative surgical workload.

**Table 4:** Primary indications for secondary pars plana vitrectomy within 2 years (*N* = 96)

Primary Indication for Reoperation	Count (*n* = 96)	Percentage (%)
**Hemorrhage-related causes**	**(56)**	**(58.4%)**
- Recurrent VH alone	42	43.8%
- Combined VH and TRD	14	14.6%
**Anatomical failure**	**(40)**	**(41.7%)**
- Post-operative TRD alone	40	41.7%
**Total**	**96**	**100.0%**

Abbreviations: VH, vitreous hemorrhage; TRD, tractional retinal detachment

## Discussion

In this retrospective cohort study of 735 PDR patients, we characterized the 24-month clinical course of post-operative VH using a 24-month survival analysis. Our findings reveal that post-operative VH is not merely a transient early complication but a persistent risk that significantly compromises long-term visual recovery and drives the majority of secondary surgical interventions. Most importantly, we observed a robust, 24-month protective association with preoperative anti-VEGF injection, which is consistent with a potential pathway independent of anatomical success.

### The role of anti-VEGF: from acute de-bulking to long-term vascular quiescence

A pivotal finding of our study is the 63% reduction in VH hazard (HR 0.37) associated with preoperative anti-VEGF therapy, a protective effect that persisted throughout the 24-month horizon. While previous literature primarily emphasizes the perioperative benefits of anti-VEGF—such as facilitating membrane dissection and minimizing intraoperative bleeding [17]—our long-term data suggest a potential sustained pharmacological association.

A critical paradox in clinical practice is how a single preoperative injection, with a vitreous half-life typically measured in weeks, can confer protection for up to two years. We propose that this sustained benefit is not a result of prolonged drug presence, but rather a consequence of long-term morphological remodeling of the retinal microvasculature [12, 18]. Preoperative VEGF inhibition induces the “pruning” and regression of fragile, active neovascularization, converting engorged, thin-walled vessels into non-functional, fibrotic stalks [10, 19, 20]. This pharmacological “pre-conditioning” significantly mitigates iatrogenic mechanical trauma to the retinal vessels during surgical manipulation, thereby preventing the formation of occult micro-vascular stumps that often serve as the source of late-onset recurrent hemorrhage.

### Decoupling pharmacological efficacy from surgical maneuvers and selection bias

To address whether these observed benefits were merely artifacts of clinical decision-making or surgical thoroughness, we performed a multidimensional validation. First, we confirmed that baseline traction severity did not skew the clinical decision to administer anti-VEGF (χ 2 = 1.09,*P* = 0.581), ensuring that our treatment groups were well-balanced regarding anatomical complexity. Second, the reviewer rightly points out that “full release of traction” is the fundamental goal of vitrectomy. By utilizing post-operative TRD as a proximal marker, we noted that macroscopic anatomical failure itself was not an independent predictor of long-term VH (*P* = 0.913), while the protective trend of anti-VEGF remained robust. However, this finding must be interpreted with substantial caution. Post-operative TRD is a multi-factorial anatomical endpoint and serves as an incomplete surrogate for overall surgical thoroughness. The lack of statistical association between TRD and VH does not imply that intraoperative factors are negligible. In clinical reality, post-operative TRD fails to capture subtle yet vital surgical variables, such as the volume of microscopic residual fibrovascular tissue, the occurrence of minor intraoperative bleeding, the precise density and placement of endolaser photocoagulation, or intraoperative visualization quality driven by media opacities. Therefore, while our model suggests that anti-VEGF provides a generalized benefit, it works in tandem with, rather than independent of, these granular surgical nuances. Finally, our interaction analysis confirmed that the “vascular buffer” provided by anti-VEGF remained stable across all traction grades (interaction *P* > 0.05). This suggests that anti-VEGF may exert a generalized pharmacological effect related to microvascular stabilization.

### The “Vascular Buffer” and the disruption of the pro-angiogenic cycle

Our sensitivity analysis using the “pure hemorrhage model” (HR = 0.32 in the TRD-excluded subgroup) provides further evidence that this protection is independent of anatomical success. Even in eyes that achieved complete traction relief and retinal reattachment, those without preoperative anti-VEGF remained at a significantly higher risk of bleeding.

This supports the hypothesis that anti-VEGF therapy may favor a long-term “vascular buffer” by potentially modifying the pro-angiogenic microenvironment [12]. By reducing residual intraocular blood—which is itself a potent pro-inflammatory and pro-angiogenic stimulus—anti-VEGF appears to help disrupt the “hemorrhage-inflammation-neovascularization” vicious cycle [21, 22], maintaining long-term microvascular quiescence regardless of whether the initial surgical indication was tractional or exudative.

### Systemic and ocular risk factors

Our multivariate analysis identified younger age, elevated HbA1c, and high serum creatinine as independent systemic predictors of post-operative VH [5, 12, 23–26]. Younger patients often present with more aggressive fibrovascular proliferation, potentially driven by higher levels of growth factors [5, 26, 27]. The associations with HbA1c and creatinine underscore the critical role of the systemic microenvironment; poor glycemic control and renal impairment likely reflect a state of chronic endothelial dysfunction and increased vascular permeability, which predisposes the eye to recurrent bleeding even after successful traction relief [25, 26]. Furthermore, severe preoperative fibrovascular traction nearly doubled the risk of VH [12, 24]. This suggests that in eyes with advanced proliferative disease, the mechanical complexity of the index surgery may leave behind a more pro-angiogenic environment, necessitating even more vigilant perioperative management.

### The burden of reoperation: a paradigm shift in post-operative PDR management

The most compelling evidence of the clinical impact of post-operative VH lies in its disproportionate contribution to the secondary surgical burden. Our analysis reveals a staggering finding: vitreous hemorrhage-related complications accounted for 58.4% of all secondary surgical interventions within the 24-month follow-up period. This indicates that more than half of the revisional surgical workload in PDR management is driven not by anatomical failure, but by vascular instability. The reoperation rate in the VH group was more than four times higher than that of the non-VH group (31.3% vs. 7.2%), highlighting a profound disparity in resource utilization and patient morbidity.

Crucially, this surgical burden is not uniformly distributed across the post-operative timeline. Our data underscores a critical distinction between the clinical courses of early and late VH. While early recurrence (≤ 1 month) often reflects transient perioperative factors and exhibited a relatively low reoperation rate (11.1%), late VH (> 1 month) emerged as the primary driver of long-term morbidity. These late-onset events were associated with a significantly higher reoperation rate (32.4%) and poorer functional recovery, representing the “lion’s share” of the revisional workload. Importantly, the potent protective effect of anti-VEGF was most pronounced against these late events, suggesting that the primary value of preoperative injection lies in maintaining long-term microvascular quiescence rather than merely managing transient perioperative oozing.

This shift in evidence demands a re-evaluation of surgical success. Historically, vitreoretinal surgeons have prioritized “anatomical success”—the relief of traction and retinal reattachment—as the gold standard of PDR surgery [28]. However, our findings challenge this traditional hierarchy. While TRD remains a grave complication, it is the persistent or recurrent hemorrhage that constitutes the “lion’s share” of the long-term surgical attrition. This shift in perspective is vital: it suggests that a surgery can be an anatomical “success” yet a clinical “failure” if the retinal microvasculature remains prone to bleeding.

From a public health and socioeconomic perspective, the implications are substantial. Each reoperation represents not only an escalation in healthcare costs and a strain on surgical facilities but also a significant psychological and functional setback for the patient [29, 30]. Our study notes that VH-related indications comprised 58.4% of all secondary operations, suggesting that optimizing perioperative management to mitigate recurrent hemorrhage could meaningfully lower the subsequent revisional burden on healthcare systems. This identifies preoperative anti-VEGF therapy and stringent systemic metabolic control not merely as “adjuncts,” but as essential strategies for enhancing the cost-effectiveness and long-term viability of PDR surgery. By stabilizing the vascular environment, we do more than clear the vitreous; we may help mitigate the cumulative “surgical burden” on the healthcare system.

### Strengths and limitations

The strengths of this study include its large sample size, the standardized 24-month survival analysis, and the use of sensitivity models to isolate vascular mechanisms.

However, several limitations must be acknowledged. First, as a retrospective study, the administration of preoperative anti-VEGF was not randomized, which introduces potential selection bias. For instance, surgeons might theoretically favor injections for eyes with more active neovascularization. To rigorously mitigate the impact of this bias, we executed a three-tier validation analysis. Our results demonstrated that baseline characteristics were well-balanced between groups, the drug’s efficacy was consistent across all traction severities, and the protective effect remained robust in subgroup analyses. These findings collectively suggest that the observed clinical benefit is pharmacologically driven rather than a mere artifact of case selection. Nevertheless, future randomized controlled trials remain the gold standard to fully eliminate such confounding factors.

Second, due to the retrospective nature of our electronic database, several critical systemic covariates were not uniformly recorded, representing a meaningful limitation that warrants explicit emphasis given our focus on post-operative hemorrhagic outcomes. Most notably, we lacked granular data on: (1) diabetes classification (Type 1 vs. Type 2), which delineates distinct systemic vascular phenotypes and metabolic trajectories; (2) urinary albumin or proteinuria markers, which serve as the pathophysiological gold standard for systemic endothelial leakiness and advanced microangiopathy; and (3) the longitudinal use of antiplatelet or anticoagulant medications, which directly modulates systemic hemostasis and intraocular re-bleeding dynamics. The absence of these variables is particularly relevant to our primary endpoint, as antithrombotic therapies can substantially exacerbate post-vitrectomy oozing from fragile vascular stumps, while active proteinuria is a much more sensitive reflector of generalized systemic capillary breakdown than gross renal clearance. Although we attempted to statistically control for the systemic microenvironment by adjusting for HbA1c and serum creatinine, these macro-markers cannot fully substitute for the specific pathophysiological insights provided by coagulation profiles or microalbuminuria. Consequently, the residual confounding stems from these omitted variables, and our multivariate Cox models may underestimate the true impact of systemic microvascular permeability and hematological homeostasis on long-term vitreous hemorrhage recurrence. This limitation necessitates a more conservative interpretation of our independent risk profiles until verified by prospectively designed protocols.

Third, a notable limitation in our methodological approach is the reliance on post-operative TRD as a surrogate for ‘incomplete traction release’ or ‘surgical thoroughness.’ Although this regression analysis helps mathematically adjust for major anatomical outcomes, post-operative TRD represents a highly simplified macroscopic category. It is incapable of capturing the full spectrum of surgical complexity and intraoperative quality, such as the exact completeness of peripheral vitreous shaving, the potential persistence of occult fibrovascular stalks, instances of self-limiting intraoperative hemorrhage, the total burning energy or precise area of endolaser treatment, and variations in visualization quality during dissection. Consequently, our ability to entirely decouple the pharmacological mechanisms of anti-VEGF from the precise technical execution of the vitrectomy remains limited by these unmeasured surgical variables.

Additionally, we must acknowledge that the discrimination of our multivariate Cox models was moderate rather than strong (C-index values of 0.68 and 0.696). While these values are entirely acceptable for establishing independent risk factors in a complex systemic disease like PDR, they do not imply high individual predictive accuracy. Consequently, these models should be used for population-level risk stratification rather than precise individual prognostic forecasting. Furthermore, other unmeasured clinical variables, such as blood pressure variability, acute postoperative glycemic spikes, or patient adherence to follow-up schedules, might also introduce unmeasured variance to long-term recurrence. Despite these constraints, this study provides one of the largest longitudinal cohorts to date evaluating the long-term surgical burden and vascular stability in PDR surgery, offering valuable evidence for perioperative optimization.

## Conclusion

In conclusion, post-operative VH is a primary driver of long-term visual impairment and healthcare resource utilization in PDR management. Preoperative anti-VEGF therapy exhibits a robust, sustained protective association, which is consistent with the hypothesis of microvascular stabilization independent of anatomical outcomes. Given that VH-related indications accounted for 58.4% of secondary surgical interventions, the implementation of preoperative anti-VEGF combined with stringent systemic metabolic control represents a targeted strategy that could meaningfully decrease the reoperation burden, offering a potentially cost-effective approach to managing the surgical workload in PDR care.

## Supplementary Information

Below is the link to the electronic supplementary material.

Supplementary Material 1

Supplementary Material 2

Supplementary Material 3

Supplementary Material 4

Supplementary Material 5: Supplementary Figure 1. Kaplan-Meier estimates of the cumulative incidence of post-operative vitreous hemorrhage between the Conbercept and Ranibizumab subgroups. The overlapping curves and the non-significant Log-rank P-value (0.675) indicate comparable long-term protective efficacy between the two anti-VEGF agents.

Supplementary Material 6: Supplementary Figure 2. Distribution of Preoperative Anti-VEGF Treatment Across Traction Severity Grades. The stacked bar chart illustrates the proportion of patients who received preoperative anti-VEGF injections compared to those who did not, stratified by preoperative traction severity: No Traction, Mild/Moderate and Severe. Pearson’s chi-squared test confirms that the clinical decision-making process for anti-VEGF administration was balanced across groups (χ 2 =1.085,P=0.581), indicating no significant selection bias based on anatomical complexity.

## Data Availability

No datasets were generated or analysed during the current study.

## Abbreviations

HR: Hazard ratio
CI: Confidence interval
Anti-VEGF: Anti-vascular endothelial growth factor
HbA1c: Glycated hemoglobin
