# Supplementary material for: Preoperative anti-VEGF and the cumulative risk of post-operative vitreous hemorrhage in PDR: a 2-year survival analysis and evaluation of surgical burden
Source: Int J Retina Vitreous. 2026 May 29;12:103. doi: 10.1186/s40942-026-00871-w (PMC13430745; doi:10.1186/s40942-026-00871-w)
Supplement: Supplementary file 1 — Supplementary Material 1 [file 40942_2026_871_MOESM1_ESM.docx]

**Supplementary Table 1. Subgroup Analysis Comparing Baseline Characteristics and Post-operative Vitreous Hemorrhage Risk Between Conbercept and Ranibizumab (n = 590).**

| **Characteristics** | **Conbercept (n = 317)** | **Ranibizumab (n = 273)** | **P-value** |
| --- | --- | --- | --- |
| **Part A: Baseline Characteristics** |  |  |  |
| Age (years), mean ± SD | 53.17 ± 9.46 | 52.67 ± 9.91 | 0.534 |
| HbA1c (%), mean ± SD | 7.21 ± 1.49 | 7.47 ±1.66 | 0.095 |
| Traction Grade, n (%) |  |  | 0.693 |
| No traction | 81 (25.6%) | 65 (23.8%) |  |
| Mild or moderate | 127 (40.1%) | 105 (38.5%) |  |
| Severe | 109 (34.4%) | 103 (37.7%) |  |
| **Part B: Multivariate Cox Regression** | **Hazard Ratio (HR)** | **95% CI** | **P-value** |
| **Drug Type** |  |  |  |
| Conbercept | Reference | - | - |
| Ranibizumab | **0.894** | **0.618 – 1.295** | **0.553** |
| Age | 0.982 | 0.964 – 1.002 | 0.071 |
| HbA1c | 1.055 | 0.938 – 1.186 | 0.374 |
| Creatinine | 1.001 | 0.999 – 1.004 | 0.207 |
| Traction Grade (Ref: No traction) |  |  |  |
| Mild or moderate | 0.824 | 0.470 – 1.445 | 0.500 |
| Severe | 1.923 | 1.162 – 3.182 | **0.011** |

Notes: Part A: Continuous variables are presented as mean ± standard deviation (SD). P-values were calculated using the independent-sample t-test. Categorical variables are presented as n (%) and compared using the Chi-squared test. Part B: Multivariate Cox proportional hazards model was adjusted for age, HbA1c, serum creatinine, and preoperative traction grade. Abbreviations: VH, vitreous hemorrhage; HbA1c, glycated hemoglobin; HR, hazard ratio; CI, confidence interval; Ref, reference.
