# Supplementary material for: Preoperative anti-VEGF and the cumulative risk of post-operative vitreous hemorrhage in PDR: a 2-year survival analysis and evaluation of surgical burden
Source: Int J Retina Vitreous. 2026 May 29;12:103. doi: 10.1186/s40942-026-00871-w (PMC13430745; doi:10.1186/s40942-026-00871-w)
Supplement: Supplementary file 2 — Supplementary Material 2 [file 40942_2026_871_MOESM2_ESM.docx]

**Supplementary Table 2. Evaluation of Potential Selection Bias and Treatment Consistency of Preoperative Anti-VEGF Therapy.**

| **Part A:Baseline Characteristics** | | | |
| --- | --- | --- | --- |
| **Characteristics** | **No Anti-VEGF (n=145)** | **Anti-VEGF (n=590)** | **P-value** |
| Traction Grade, n (%) |  |  | 0.581 |
| No traction | 30 (20.7%) | 146 (24.7%) |  |
| Mild or moderate | 59 (40.7%) | 232 (39.3%) |  |
| Severe | 56 (38.6%) | 212 (35.9%) |  |
| **Age, mean (SD), y** | 51.86 (9.92) | 52.94 (9.67) | 0.233 |
| **Sex, male, n. (%)** | 90(62.1%) | 391(66.3%) | 0.392 |
| History of diabetes**, mean (SD), y** | 8.27 (5.97) | 8.69 (6.21) | 0.465 |
| HbA1c, mean (SD),% | 7.58 (1.69) | 7.34 (1.58) | 0.098 |
| **Creatinine**, mean (SD), umol/L | 103.34 (40.90) | 105.35 (68.73) | 0.735 |
| Previous laser**, No. (%)** | 46 (31.7%) | 192 (32.5%) | 0.929 |
| Surgical Indication, n (%) |  |  | 0.645 |
| VH | 86(59.3%) | 365 (61.9%) |  |
| TRD | 6 (4.1%) | 31 (5.3%) |  |
| TRD+VH | 53(36.6%) | 194 (32.9%) |  |
| **Part B:Interaction Analysis of Preoperative Anti-VEGF and Traction Grade** | | | |
| **Variable** | **Hazard Ratio (HR)** | **95% CI** | **P-value** |
| **Main Effects** |  |  |  |
| Preoperative Anti-VEGF | 0.44 | 0.19 – 1.00 | 0.050 |
| Traction Grade:Mild or moderate | 1.55 | 0.70 – 3.43 | 0.279 |
| Traction Grade:Severe | 1.91 | 0.86 – 4.24 | 0.111 |
| **Interaction Terms** |  |  |  |
| Anti-VEGF * Traction Grade:Mild or moderate | 0.58 | 0.22 – 1.52 | 0.267 |
| Anti-VEGF * Traction Grade:Severe | 1.05 | 0.41 – 2.66 | 0.926 |
| **Part C:Subgroup Analysis: Anti-VEGF Efficacy by Traction Grade** | | | |
| ****Subgroup (Traction)**** | **Hazard Ratio (HR)** | **95% CI** | **P-value** |
| No traction | 0.53 | 0.24 – 1.20 | 0.129 |
| Mild or moderate | 0.26 | 0.16 – 0.45 | **< 0.001** |
| Severe | 0.45 | 0.29 – 0.71 | **< 0.001** |

Notes: Part A: Baseline characteristics compared between eyes that received preoperative anti-VEGF and those that did not. P-values were calculated using Independent-sample t-tests for continuous variables, and Chi-squared or Fisher’s exact tests for categorical variables. Part B: Interaction terms were incorporated into the multivariate Cox proportional hazards model to assess whether the treatment effect was modified by disease severity. Part C: Subgroup HRs represent the risk of post-operative VH for the anti-VEGF group relative to the control group within each traction level, adjusted for age and HbA1c.

Abbreviations: VH, vitreous hemorrhage; TRD, tractional retinal detachment; HR, hazard ratio; CI, confidence interval; SD, standard deviation; HbA1c, glycated hemoglobin.
