# Supplementary material for: Preoperative anti-VEGF and the cumulative risk of post-operative vitreous hemorrhage in PDR: a 2-year survival analysis and evaluation of surgical burden
Source: Int J Retina Vitreous. 2026 May 29;12:103. doi: 10.1186/s40942-026-00871-w (PMC13430745; doi:10.1186/s40942-026-00871-w)
Supplement: Supplementary file 3 — Supplementary Material 3 [file 40942_2026_871_MOESM3_ESM.docx]

**Supplementary Table 4.Multivariate Cox Proportional Hazards Model with Interaction Terms for 2-Year Post-operative Vitreous Hemorrhage.**

| ****Variable**** | ****Hazard Ratio (HR)**** | ****95% Confidence Interval (CI)**** | ****P-value**** |
| --- | --- | --- | --- |
| **Systemic and Baseline Factors** |  |  |  |
| Age (per 10-year increase) | 0.982 | 0.968 – 0.998 | **0.023** |
| HbA1c (%) | 1.109 | 1.012 – 1.215 | **0.027** |
| Serum Creatinine (umol/L) | 1.002 | 1.001 – 1.004 | **0.010** |
| **Primary Predictors** |  |  |  |
| Preoperative Anti-VEGF (Yes vs. No) | 0.445 | 0.196 – 1.008 | 0.052 |
| Traction Grade : Mild/Moderate | 1.594 | 0.719 – 3.532 | 0.251 |
| Traction Grade : Severe | 1.826 | 0.824 – 4.047 | 0.138 |
| **Anatomical Outcome (Surgical Surrogate)** | | | |
| Post-operative TRD (Yes vs. No) | 1.031 | 0.595 – 1.786 | 0.913 |
| **Interaction Terms** |  |  |  |
| Anti-VEGF * Traction Grade:Mild or moderate | 0.549 | 0.209 – 1.442 | 0.224 |
| Anti-VEGF * Traction Grade:Severe | 1.084 | 0.427 – 2.753 | 0.866 |

**Notes:**

VH, vitreous hemorrhage; TRD, tractional retinal detachment; HbA1c, glycated hemoglobin; HR, hazard ratio; CI, confidence interval.

Post-operative TRD was incorporated into the model as a clinical surrogate for "incomplete release of vitreoretinal traction" or anatomical failure.

Interaction terms (Anti-VEGF × Traction Grade) were utilized to evaluate whether the protective efficacy of anti-VEGF therapy was modified by the severity of preoperative anatomical complexity.

Bold P-values indicate statistical significance (P<0.05).
