# Supplementary material for: Preoperative anti-VEGF and the cumulative risk of post-operative vitreous hemorrhage in PDR: a 2-year survival analysis and evaluation of surgical burden
Source: Int J Retina Vitreous. 2026 May 29;12:103. doi: 10.1186/s40942-026-00871-w (PMC13430745; doi:10.1186/s40942-026-00871-w)
Supplement: Supplementary file 4 — Supplementary Material 4 [file 40942_2026_871_MOESM4_ESM.docx]

**Supplementary Table 3. Clinical Characteristics and Anti-VEGF Efficacy Categorized by Timing of VH.**

| **Category** | **Count** | **Reoperation Rate (%)** | **Final LogMAR VA** | **OR of Anti-VEGF (95% CI)** |
| --- | --- | --- | --- | --- |
| No VH | 556 | 7.2% | 0.92 ± 0.60 | Ref |
| Early VH (≤1m) | 9 | 11.1% | 1.14 ± 0.65 | - |
| Late VH (>1m) | 170 | **32.4%** | **1.34 ± 0.75** | **0.289 (0.19-0.43)** |
| P-value | - | **< 0.001** | **< 0.001** | **< 0.001** |

**Notes:**

VH was categorized based on the timing of the first recurrent event within 24 months post-surgery.

Reoperation Rate: Calculated as the percentage of eyes requiring secondary pars plana vitrectomy within each category.

Final LogMAR VA: Best-corrected visual acuity at the final follow-up (mean ± standard deviation).

OR of Anti-VEGF: Odds ratios (95% confidence intervals) for the occurrence of VH in each category, calculated using Fisher’s exact test.

Ref: Reference group. The symbol "—" for Early VH indicates that the sample size (n=9) was insufficient for a robust OR calculation.

P-values: Compare differences across the three groups using the Kruskal-Wallis test for continuous variables and the Chi-squared test (or Fisher's exact test) for categorical variables. Abbreviations: VH, vitreous hemorrhage; LogMAR, logarithm of the minimum angle of resolution; VA, visual acuity; OR, odds ratio; CI, confidence interval.
