# Supplementary figures and images for: Preoperative anti-VEGF and the cumulative risk of post-operative vitreous hemorrhage in PDR: a 2-year survival analysis and evaluation of surgical burden
Source: Int J Retina Vitreous. 2026 May 29;12:103. doi: 10.1186/s40942-026-00871-w (PMC13430745; doi:10.1186/s40942-026-00871-w)

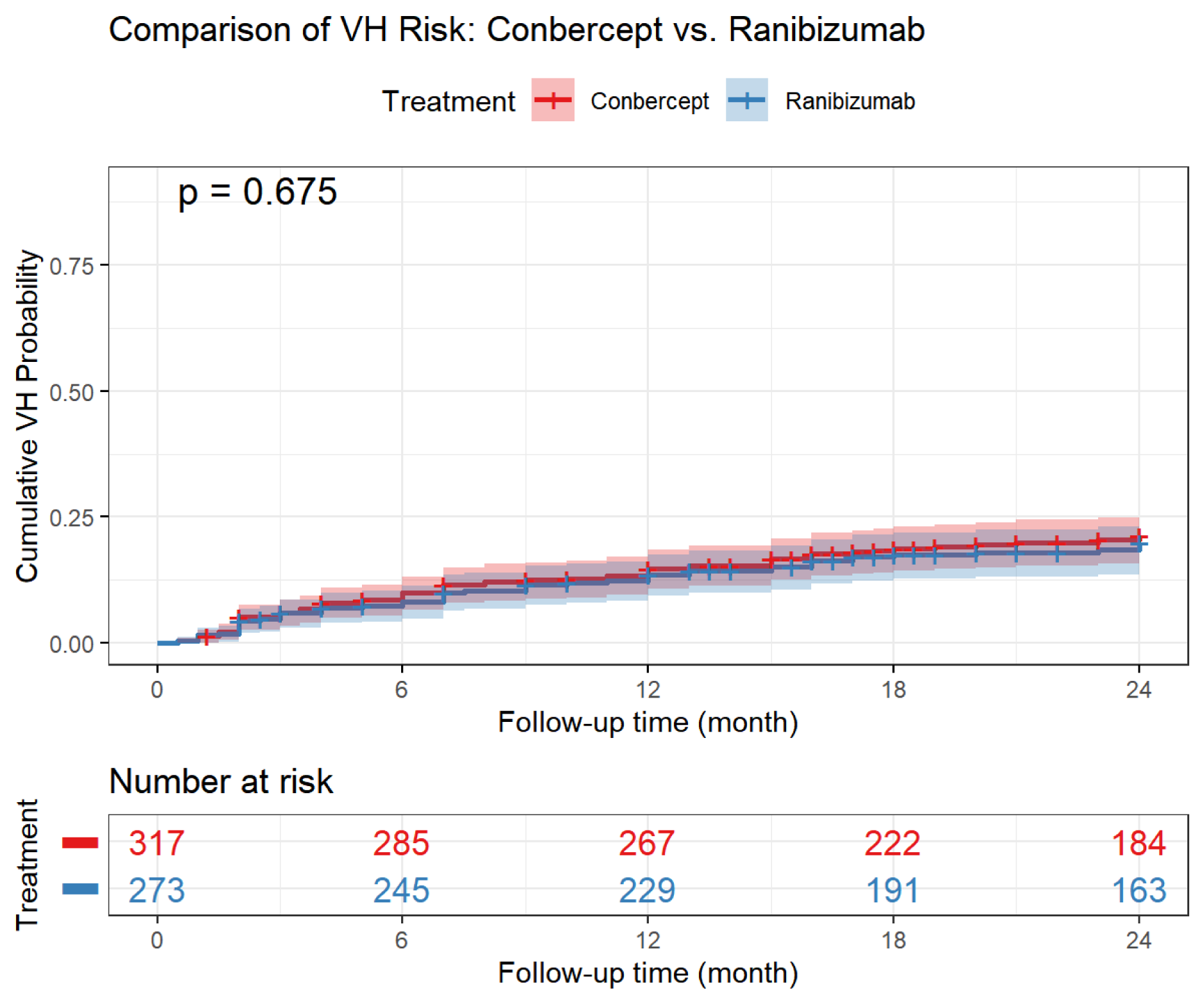

Supplement: Supplementary file 5 — Supplementary Material 5: Supplementary Figure 1. Kaplan-Meier estimates of the cumulative incidence of post-operative vitreous hemorrhage between the Conbercept and Ranibizumab subgroups. The overlapping curves and the non-significant Log-rank P-value (0.675) indicate comparable long-term protective efficacy between the two anti-VEGF agents. [file 40942_2026_871_MOESM5_ESM.tif]

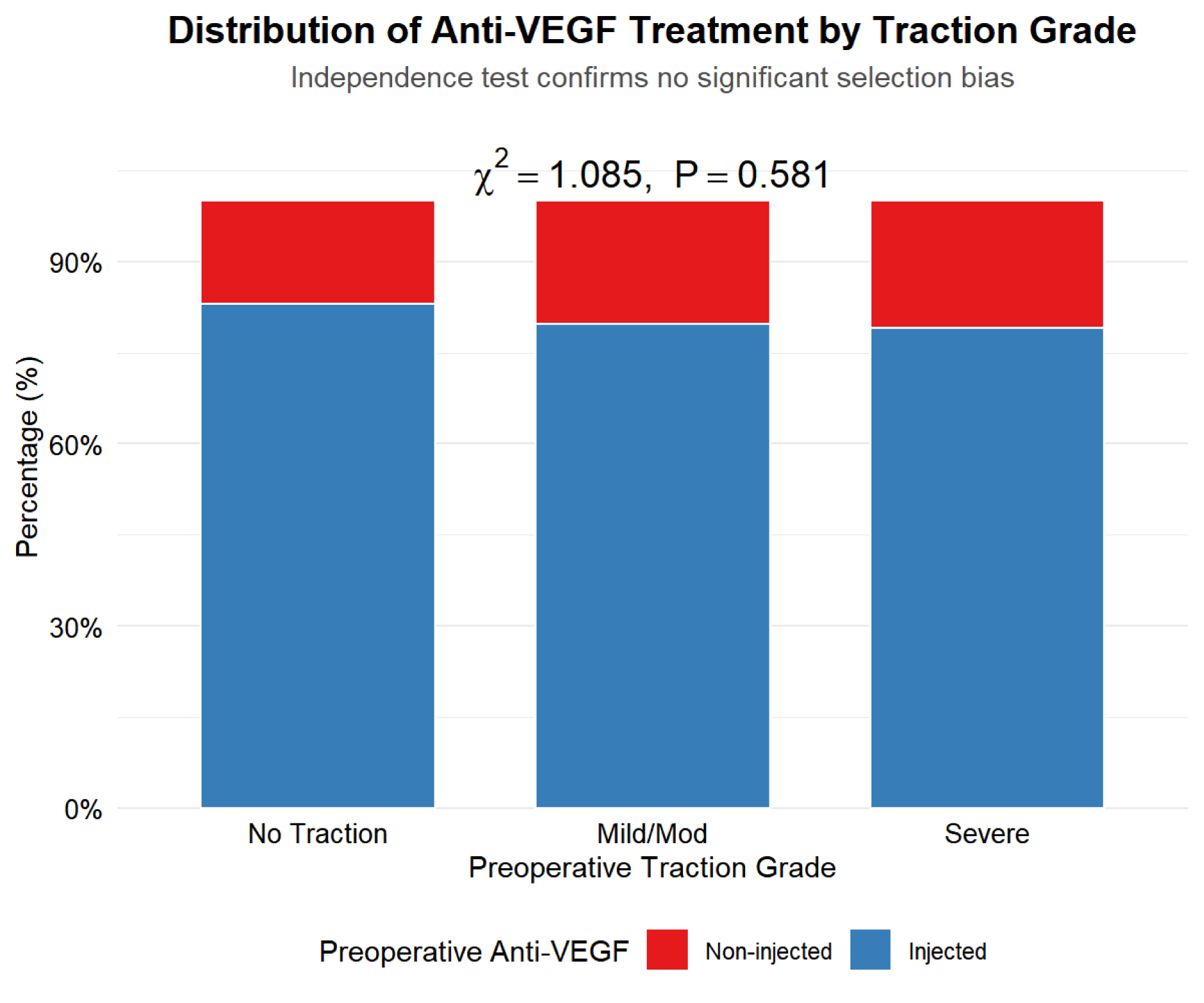

Supplement: Supplementary file 6 — Supplementary Material 6: Supplementary Figure 2. Distribution of Preoperative Anti-VEGF Treatment Across Traction Severity Grades. The stacked bar chart illustrates the proportion of patients who received preoperative anti-VEGF injections compared to those who did not, stratified by preoperative traction severity: No Traction, Mild/Moderate and Severe. Pearson’s chi-squared test confirms that the clinical decision-making process for anti-VEGF administration was balanced across groups (χ 2 =1.085,P=0.581), indicating no significant selection bias based on anatomical complexity. [file 40942_2026_871_MOESM6_ESM.tif]
